# Radioguided surgery with *β* radiation: a novel application with Ga^68^

**DOI:** 10.1038/s41598-018-34626-x

**Published:** 2018-11-01

**Authors:** Francesco Collamati, Valerio Bocci, Paolo Castellucci, Micol De Simoni, Stefano Fanti, Riccardo Faccini, Alessandro Giordano, Daria Maccora, Carlo Mancini-Terracciano, Michela Marafini, Riccardo Mirabelli, Silvio Morganti, Riccardo Schiavina, Teresa Scotognella, Giacomo Traini, Elena Solfaroli Camillocci

**Affiliations:** 1 Istituto Nazionale di Fisica Nucleare, Sezione di Roma, Roma, Italy; 2 Dipartimento di Fisica, Sapienza Università di Roma, Roma, Italy; 3 Dip. Scienze di Base e Applicate per l’Ingegneria, Sapienza Università di Roma, Roma, Italy; 4 Fondazione Policlinico A. Gemelli IRCCS - Università Cattolica Sacro Cuore, Rome, Italy; 5 Department of Urology, University of Bologna, Bologna, Italy; 6 Fondazione Policlinico Universitario A. Gemelli IRCCS, Rome, Italy; 7 Medicina Nucleare Metropolitana, Bld 30, AOU Policlinico S. Orsola-Malpighi, Bologna, Italy; 8 Scuola di Specializzazione in Fisica Medica, Sapienza Università di Roma, Roma, Italy; 9 Museo Storico della Fisica e Centro Studi e Ricerche E.Fermi, Roma, Italy

## Abstract

Radio Guided Surgery is a technique helping the surgeon in the resection of tumors: a radiolabeled tracer is administered to the patient before surgery and then the surgeon evaluates the completeness of the resection with a handheld detector sensitive to emitted radiation. Established methods rely on *γ* emitting tracers coupled with *γ* detecting probes. The efficacy of this technique is however hindered by the high penetration of *γ* radiation, limiting its applicability to low background conditions. To overtake such limitations, a novel approach to RGS has been proposed, relying on *β*^−^ emitting isotopes together with a dedicated *β* probe. This technique has been proved to be effective in first *ex*-*vivo* trials. We discuss in this paper the possibility to extend its application cases to ^68^Ga, a *β*^+^ emitting isotope widely used today in nuclear medicine. To this aim, a retrospective study on 45 prostatic cancer patients was performed, analysing their ^68^Ga-PSMA PET images to asses if the molecule uptake is enough to apply this technique. Despite the expected variability both in terms of SUV (median 4.1, IQR 3.0–6.1) and TNR (median 9.4, IQR 5.2–14.6), the majority of cases have been found to be compatible with *β*-RGS with reasonable injected activity and probing time (5 s).

## Introduction

Radioguided surgery (RGS) is a technique helping the surgeon to achieve a resection of the tumor as complete and precise as possible. In this technique, a radiolabeled tracer, that is preferentially taken up by the tumor, is administered to the patient before the surgery. During the procedure itself, the surgeon is then provided with an handheld detector (named *probe*) that, being sensitive to radiation emitted by the tracer, allows to recognise areas of high uptake of the molecule, thus suggesting the possible presence and extension of the tumoral cells.

Established methods use nowadays a combination of a *γ*-emitting tracer with a *γ*-radiation-detecting probe^1,2^. However, since *γ* radiation can penetrate large amounts of tissue, any uptake of the tracer in nearby healthy tissue represents a non-negligible background, that often limits or eventually prevents the use of this technique.

To overcome these limits and extend the range of applicability of RGS, it was suggested^3^ to use pure *β* ^−^emitting radio-isotopes instead of *γ*-emitting tracers. In fact, ${\beta }^{-}$ radiation penetrates only few millimetres of tissue with essentially no $\gamma$ contamination, since the *bremsstrahlung* contribution, that has a 0.1% emission probability at $\beta$-product energies, can be considered negligible^4^. Such a technique would allow a clearer delineation of margins of the lesioned tissue together with a substantial reduction (due to low penetration of ${\beta }^{-}$ particles) of the dose given to the medical staff. A proper detector has been developed to test the technique. This ${\beta }^{-}$ probe is characterised by high sensitivity to $\beta$ particles, whilst being substantially transparent to $\gamma$ contamination. In the recent years, this proposed novel technique of RGS has been proved not only to be theoretically possible^5,6^, but also its actual feasibility has been demonstrated with *ex*-*vivo* tests on meningioma samples marked with ^90^Y-DOTATOC^7^.

Despite the success of these first clinical trials, the fact that basically only somatostatin analogues can currently be marked with a pure ${\beta }^{-}$ emitter (^90^Y) poses a strong limitation to the possible applications of this technique. To this purpose, a systematic study of the detector performances with several radioisotopes (mainly focusing on “non pure” ${\beta }^{-}$ emitters) has been performed^8^. In this study, also ^18^F was considered, due to the huge amount of application cases the extension to such an isotope would represent. However, it turned out that at the current status, the *β* probe does not allow an efficient detection of the emitted radiation, its efficacy being limited mainly by the low energy endpoint of emitted *β*^+^ particles, rather than by the copious background of annihilation photons, for which the probe is essentially transparent.

In this context, ^68^Ga could in principle be an interesting isotope for an extention of the technique. It is in fact another widespread ${\beta }^{+}$ emitting isotope, commonly used in many fields of oncologic applications, not only in the diagnosis of Neuroendocrine Tumors (NET) labelled with different peptides such as DOTA-NOC, DOTA-TOC, DOTA-TATE^9^ but also, more recently, with the advent of Ga-labelled PSMA compounds such as PSMA 11 and PSMA 617^10^. The main difference with respect to ^18^F is represented by the much higher energy end point for the $\beta$ particle, that is of 1.899 MeV (to be compared to $0.633\,{\rm{MeV}}$ of ^18^F and to $2.280\,{\rm{MeV}}$ of the ${\beta }^{-}$ spectrum of ^90^Y). The beta energy endpoint of ^68^Ga is thus similar to the one of ^90^Y, whilst the unavoidable *γ* background due to positron annihilation holds as a substantial difference between the two cases. However, in presence of favourable conditions in terms of both absolute and relative to the healthy tissue uptake of the tumor, the low sensitivity of the probe to photons could allow to use *β*-RGS also with ^68^Ga.

All in all, the main concern with the abundant $\gamma$ emission would be represented by radio-protection issues of the medical personnel and the patient, for which the same considerations standing for currently used $\gamma$-RGS hold. However, the high sensitivity of the detector to positrons allows to reduce the activity to inject to the patient, and thus the dose given to the personnel. On the other side, the dose to the patient would be comparable to a PET scan.

The aim of this study is thus to evaluate the feasibility of such a technique in a first application case involving ^68^Ga besides somatostatin analogues, namely lymphoadenectomy in case of prostatic surgery with ^68^Ga-PSMA.

In the last few years, PSMA PET has emerged as an accurate tool to detect metastatic lymph nodes (LNs), especially in patients with high risk for distant involvement at presentation or in patients showing Biochemical Recurrence (BCR) of prostate cancer, candidates or eligible for salvage lymphadenectomy^11^. In both primary staging or in salvage surgery procedures in case of BCR the localisation of small lymph nodes may result difficult for surgeons, especially if the lymph nodes are located in atypical sites. The use of Radio Guided Surgery may help surgeons in the detection of such small metastatic lymph nodes with the aim of increasing accuracy, reducing the operation time and improving oncological outcome^12^.

The use of ^68^Ga-PSMA as radiopharmaceutical has the advantage to allow at the same time preoperative imaging using PET and radio surgical guidance using dedicated beta probes. In this paper we have studied retrospectively PET scans of patients with prostate cancer after administration of ^68^Ga-PSMA. Following the procedure in ref.^5^, we use the measured uptake in tumor and nearby healthy tissues to evaluate the sensitivity of a probe, designed for $\beta$ radiation^13^, to the signals of interest, thus evaluating the feasibility of the application of RGS in this case.

## Materials and Methods

### Patients selection

Since the aim of this study is to evaluate the feasibility of $\beta$-RGS in patients with prostatic cancer showing increased uptake for ^68^Ga-PSMA-11 (HBED-CC) (hereinafter called simply “^68^Ga-PSMA”) at PET/CT investigation, we selected 45 patients with a positive exam performed either for staging or restaging in case of relapse (1 patient was T1N0M0, 12 pts were T2N0M0, 5 were T2N1M0, 21 pts were T3N0M0, 6 were T3N1M0). PET/CT scans were performed at Policlinico Sant’Orsola Malpighi in Bologna following standard acquisition methods. We collected data for 58 different lesions, either in the prostatic gland or other lymph nodal localisations. All patients gave written informed consent to participate in the clinical research. The study, as part of a larger trial on prostate cancer imaging with PET^14^, has been approved by the Ethics Committee of the Policlinico Sant’Orsola Malpighi. Moreover, this research is compliant with applicable Italian law and with the Charter of Fundamental Rights of the European Union (in particular with Art. 3 and Art. 8), the Declaration of Helsinki, the Oviedo Bioethics Convention and the European Union Regulation N. 536/2014 of the European Parliament and of the Council on clinical trials on medicinal products for human use, and repealing Directive 2001/20/EC.

### Estimate of the PSMA Uptake

To quantify the applicability of this technique, it is necessary to know the SUV and TNR of PSMA in prostatic cancer.

The Standardised Uptake Value (SUV) is a parameter used to take into account the differences in administered activity of tracer injected to the patients, and is defined as:

1 $${\rm{SUV}}=\frac{\mu \,W}{{A}_{adm}\,{e}^{-0.693{\rm{\Delta }}{t}_{PET}/{T}_{Ga}^{1/2}}},$$

where *μ* is the mean value of PET activity of the considered point, *A*_*adm*_ is the administered activity, *W* mass of the patient, ${\rm{\Delta }}{t}_{PET}$ is the time elapsed between the administration and the PET scan, and ${T}_{Ga}^{\mathrm{1/2}}$ is the ^68^Ga half-life.

The Tumor Non tumor Ratio (TNR) is defined as the ratio between the SUVs of the tumor and of the nearby healthy tissue.

Keeping in mind the application case of this study, that is the intraoperative discrimination of tumor remnants and/or positive lymph nodes during prostatectomy, we decided to choose as reference “healthy tissue” the area around the active lesion of interest, rather than for example a healthy lymph-node with a generic localisation. It is in fact this one the area the surgeon must be able to eventually distinguish from the lesion in a hypothetical application case.

To obtain these uptakes, we examined ^68^Ga-PSMA PET scans of the patients in the cohort. Patients were injected with a 2 MBq/kg activity of ^68^Ga-PSMA. After a 60-min uptake period, whole-body imaging was performed on a bismuth germanium oxide equipped Discovery 690 PET/CT scanner (GE Healthcare) using a standard vertex-to-pelvis protocol. The CT acquisition protocol included low- dose CT (120 kV, automatic amperage, noise index 25, pitch of 1.375, and 3.75-mm slice thickness) for attenuation correction followed by a whole-body PET scan (3 min per bed position). The PET scans were acquired in 3-dimensional mode in a 256 256 matrix (voxel size, 2.73 · 2.73 · 3.17 mm). Images were reconstructed using VUE Point HD (GE Healthcare) attenuation-weighted ordered-subsets expectation maximization (2 iterations, 16 subsets) followed by a post reconstruction smoothing gaussian filter (5 mm in full width at half maximum).

These PET images were retrospectively analysed with Advance GE workstation (version Advantage 4.6). In particular, the “3d-isocontour” tool was used to define ROIs (Regions Of Interest) throughout several slices in which to evaluate the mean uptake, thus forming VOIs (Volumes Of Interest). The threshold for this selecting tool was set to 50% of the maximum value, and the VOIs were chosen to be approximately of the order of $100\,{{\rm{mm}}}^{3}$ in volume for homogeneity of comparison.

For each VOI, the average SUV ($\overline{SUV}={\sum }_{i=1}^{N}\,SU{V}_{i}/N$, where *SUV*_*i*_ is referred to the individual *N* pixels), the SUV RMS (${{\rm{\Delta }}}_{SUV}$, defined as $\sqrt{{\sum }_{i=1}^{N}\,{(SU{V}_{i}-\overline{SUV})}^{2}/(N-1)}$) and the VOI volume (*V*) were measured. From this latter parameter, knowing the size of the voxels (*V*_*v*_), the number of voxels in the VOI has been obtained by means of:

2 $$N=\frac{V}{{V}_{v}},$$

and thus the error on the mean SUV value was calculated:

3 $${\sigma }_{\overline{SUV}}=\frac{{{\rm{\Delta }}}_{SUV}}{\sqrt{N}}.$$

In some occasions, the analysis program was not able to automatically extend the ROI through several contiguous slices. In these cases several ROIs ($i=1\ldots n$) were manually created through the slices in order to achieve the desired target volume for the VOI, measuring for each one the average SUV (${\overline{SUV}}_{i}$), the SUV rms (${{\rm{\Delta }}}_{SU{V}_{i}}$) and the ROI volume (*V*_*i*_). Then, to obtain the average value of the SUV in the VOI, a weighted average of the single mean SUVs of each ROI was performed, using as weight the inverse of the error on the mean SUV of the single ROI (obtained as in Eq. 3), according to the following formula:

4 $$\overline{SUV}=\frac{{\sum }_{i\mathrm{=1}}^{n}\frac{SU{V}_{i}}{{\sigma }_{i}^{2}}}{{\sum }_{j\mathrm{=1}}^{n}\frac{1}{{\sigma }_{j}^{2}}}\mathrm{.}$$

The error on the average SUV of the VOI is then:

5 $${\sigma }_{\overline{SUV}}=\frac{1}{\sqrt{{\sum }_{i\mathrm{=1}}^{n}\frac{1}{{\sigma }_{i}^{2}}}}\mathrm{.}$$

### Evaluation of TNR

From the average values for the SUVs in the VOIs of interest for both lesions and healthy tissues, Tumor Non-tumor Ratios (TNR) have been calculated. Calling ${\overline{SUV}}_{T}$ and ${\overline{SUV}}_{H}$ the mean SUV of the Tumor and of Healthy Tissue respectively, the TNR is calculated as:

6 $$TNR=\frac{{\overline{SUV}}_{T}}{{\overline{SUV}}_{H}}.$$

The error on the TNR is obtained combining the errors on both SUVs (${\sigma }_{{\overline{SUV}}_{T}}$ and ${\sigma }_{{\overline{SUV}}_{H}}$), according to:

7 $${\sigma }_{TNR}=TNR\sqrt{{(\frac{{\sigma }_{{\overline{SUV}}_{T}}}{{\overline{SUV}}_{T}})}^{2}+{(\frac{{\sigma }_{{\overline{SUV}}_{H}}}{{\overline{SUV}}_{H}})}^{2}}.$$

### The *β* Probe

A crucial element in the development of the proposed RGS method is the design of the detector to be used during the RGS procedure. As described in previous studies^5,6^, we developed a *β* probe, which exploits the low penetration power of *β* radiation by reducing the size of the lateral shielding, with the result of a light and handy tool, with respect to commercial *γ* probes. The active area of the detector is made of *p*-*terphenyl*, that is an organic scintillator characterised by low density and high light yield, which makes it an ideal choice to have good sensitivity to *β* particles, while being substantially transparent to photons^15^.

In particular, the actual probe prototype, which has been tested on *β*^−^ radiation in a clinical trial with meningioma *ex*-*vivo* samples^7^, is composed by a sensitive cylinder of p-terphenyl with a radius of 2.55 mm and a 3-mm depth. To maximise the accuracy on the direction of the incoming radiation, the sensitive region is screened by 3 mm of polyvinylchloride. The scintillation light is read by a Silicon Photo Multiplier sensor (SiPM, SenSL series C, mod. 10035), controlled and read out by means of the ArduSiPM electronics^16^.

It has to be noted that an open surgery approach is not necessarily an optimal use case for this technique, and indeed the final goal is to develop a detector suited for laparoscopic, eventually robotic, surgery. The design of the “robotic probe” is in fact already ongoing, using the same detection principle than the actual probe (scintillating crystal + SiPM), but encapsulated in a small, “drop in” device to be handled by laparoscopic or robotic instruments.

### Calculation of expected rates

In order to quantify the performances of the probe in the real application scenario, a benchmark surgical configuration was reproduced within a Monte Carlo (MC) simulation with the FLUKA program^17^. Two simulations have been developed. The first one reproduces the probe exposition to a small ($\sim 0.1\,{\rm{mL}}$) tumoral remnant encapsulated in healthy tissue; the second one has been developed to calculate the background contribution of *γ*s from the whole patient body. In the scintillator, 28000 optical photons per MeV of deposited energy^15^ are produced and tracked. To increase the simulation precision, the *δ* rays production threshold and the electron transport threshold have been set to 10 keV in the scintillator and in the materials in contact with it. Moreover, in these materials the electrons step size has been limited to make them loose no more than 1% of their energy per each step. The event is considered detected if a number of optical photons greater than a threshold crosses the boundary with the SiPM. Such a threshold, as the other parameters of the optical photons simulation, has been set using data from a dedicated measurement campaign. More details on the MC simulation and its optimisation can be found in^8^.

All in all, SUV and TNR retrieved from the retrospective study can be used to simulate the expected activity in the considered lesion during a possible RGS procedure. The MC code allows thus to obtain the rates, in counts per second (*cps*), we expect to count on the tumor (*R*_*T*_) and on the nearby healthy tissue (*R*_*H*_).

Obviously, such a prediction takes as an input the amount of activity injected to the patient before surgery and the time elapsed between the injection and the procedure. These parameters are in fact necessary to foresee the specific activity in the areas of interest at the time of RGS. In this study we assumed that an activity of $3\,\mathrm{MBq}/\mathrm{kg}$ of ^68^Ga-PSMA could be administered to the patient, thus using as a reference (for a $70\,{\rm{kg}}$ patient) a total activity of $210\,{\rm{MBq}}$. The difference in the activity to be administered in case of RGS, with respect to the one of the PET scans used for the retrospective study earlier described, can be taken into account by proper normalisation, assuming a linear behaviour of the expected uptakes.

The choice of the time between the injection and the procedure is instead quite complex. On one hand, in fact, there are some technical constraints regarding the need to inject the patient, to do the pre-operative PET scan (needed to assess the actual uptake for PSMA) and to safely transport the patient from Nuclear Medicine to the Operating Room. On the other hand, however, the half-life of ^68^Ga is of $68\,{\rm{\min }}$, thus implying that every hour elapsed a factor $\sim 2$ of activity is lost. Therefore, in this study we assumed that the procedure is performed $2\,{\rm{h}}30\,{\rm{\min }}$ ($150\,{\rm{\min }}$) after the injection of the radiopharmaceutical.

From the estimated ${R}_{T}$ and ${R}_{H}$ it is possible to calculate the minimal probing time (${t}_{probe}$) the probe needs to be over the target to be able to discriminate with sufficient accuracy tumor from healthy tissue.

To this aim, we applied in this study the same approach applied and described in ref.^3^, that relies on the estimation of the rate of false positive (FP) and false negative (FN) signals. For a given value of the probe acquisition time (${t}_{probe}$), the number of signal counts from the tumour and the background is distributed according to a Poisson distribution with mean ${\mu }_{T}={R}_{T}\times {t}_{probe}$ and ${\mu }_{H}={R}_{H}\times {t}_{probe}$, respectively. Given the minimum number of signal counts (*th*) needed to flag a positive identification, FP is computed as the fraction of times the background would yield a positive signal:

8 $$FP=1-\sum _{N\mathrm{=0}}^{th-1}\,{P}_{{\mu }_{H}}(N),$$

where ${P}_{\mu }(N)$ indicates the Poisson probability to have *N* if the mean is *μ*. Similarly, FN is the fraction of times a tumour residual would not yield a signal:

9 $$FN=\sum _{N=0}^{th-1}\,{P}_{{\mu }_{T}}(N).$$

To determine the minimum probing time, FN and FP are computed in a grid of ${t}_{probe}$ and *th* and the smallest value of ${t}_{probe}$ for which $FN < 5 \%$ and $FP\approx 1 \%$ is determined.

### ROC Analysis

By means of the procedure described in the previous section the optimal probing time for each lesion has been estimated. However, in the real application case, and thus during the RGS procedure, the time the surgeon will spend on each analysed spot is almost constant, and can not depend on the unknown actual activity of the lesion. First *ex*-*vivo* tests suggest that, regardless of the uptake of the sample, it is natural to spend on it at least $\approx \,3$ before being confident enough to discriminate if it is healthy or not.

Therefore, we performed a sensitivity analysis to evaluate the discriminating power of our technique, assuming a fixed probing time of ${t}_{probe}=3\,{\rm{s}}$, and varying the threshold (*th*) in terms of total number of counts in this time interval. For each threshold value, “sensitivity” and “specificity” were calculated and plotted in a ROC curve, the area of which can be used as evaluation of the discriminating power of the proposed technique.

## Results

### Uptake and TNRs

The analysis procedure described above was then applied to all the 58 lesions to obtain the respective SUVs and TNRs. Median SUV was 4.1 (InterQuartile Range 3.0–6.1), while median TNR was 9.4 (IQR 5.2–14.6).

**Figure 1:**
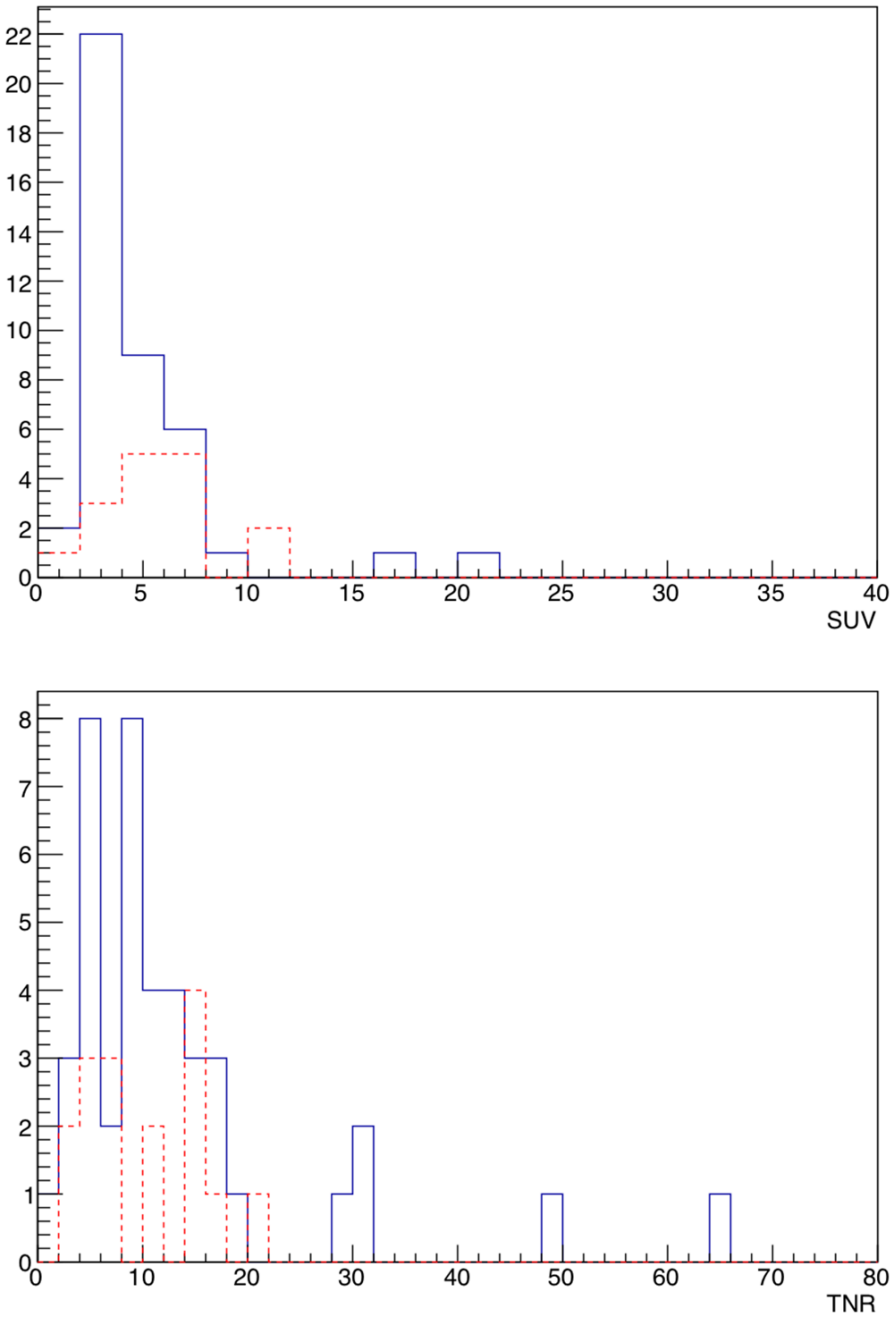
Distribution of SUVs (top) and TNRs (bottom) for all the 58 lesions. The blue (continue) line represents lymph nodes, while the red (dotted) line represents local lesions.

**Figure 2:**
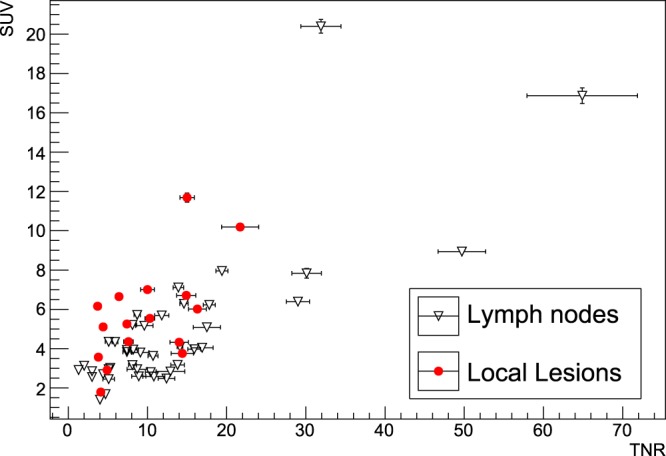
Scatter plot showing SUV as a function of TNR for all the 58 lesions, dividing between local lesions and lymph nodes. Errors shown in the bars are obtained as described in the text.

In Fig. 1 SUVs and TNRs are shown in histograms, keeping the distinction between local lesions in the prostatic situs and lymph nodes, while Fig. 2 shows the scatter plot of SUV versus TNR.

### Sensitivity Analysis

**Figure 3:**
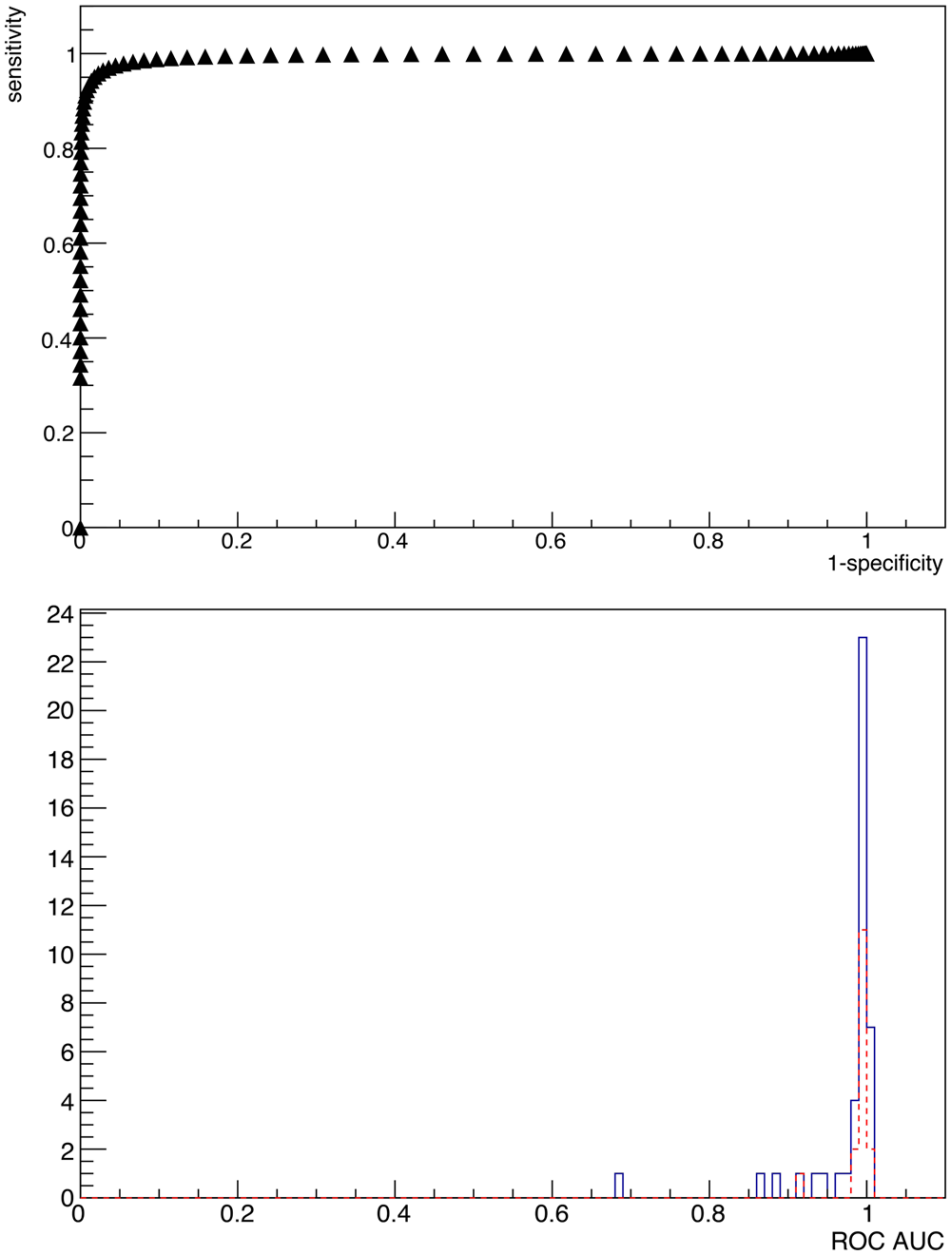
ROC curve for lymph node lesion number 1 (top), and distribution of Area Under Curve of ROCs of all the considered patient (bottom). The AUC for the shown case is 0.9951. The blue (continue) line represents lymph nodes, while the red (dotted) line represents local lesions. The probing time has been fixed to ${t}_{probe}=3\,{\rm{s}}$.

Figure 3 (top) shows the ROC curve, obtained as described previously in the text, for lymph node 1 lesion. Furthermore, Fig. 3 (bottom) shows the distribution of the Area Under Curve for all the considered 58 lesions.

### Expected Performances of RGS with *β* Decays

**Figure 4:**
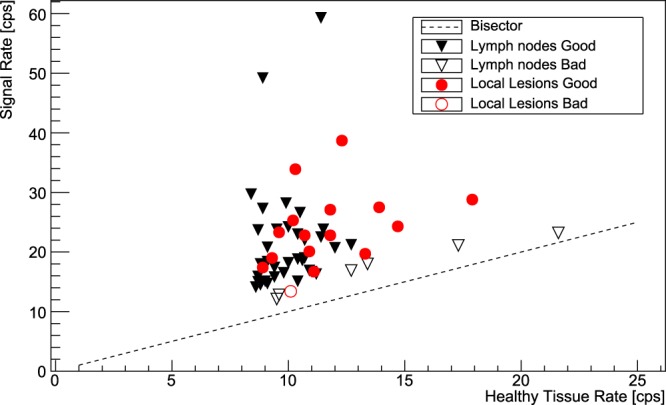
Counting rates expected on lesions (Signal) versus the ones expected on healthy tissue. The dashed line shows the bisector, representing the case in which the probe gives the exact same count over signal than over healthy tissue, having thus no sensitivity at all. In addition to the usual division among local lesions and lymph nodes, points are also classified (“Good” or “Bad”) according to the ROC sensitivity test described in the text, having fixed a probing time of 3 s and requiring $AUC > 0.95$. The rates correspond to the application case described in the text (injection of 210 MBq of ^68^Ga-PSMA $150\,{\rm{\min }}$ before the surgery).

From the results for SUVs and TNRs, shown in Figs 1 and 2, following the procedure described previously in the text, we obtained the counting rates expected in a real application scenario. The results are shown in Fig. 4, where in addition to the usual division between local lesions and lymph nodes the classification based on ROC sensitivity test is also shown (“Good” if $AUC > 0.95$, “Bad” otherwise). Here the bisector represents the “worst case”, in which lesion and healthy tissue give the same rate. Median probing time (${t}_{probe}$) was $3.0\,{\rm{s}}$ (IQR 1.5–5.5 s).

**Figure 5:**
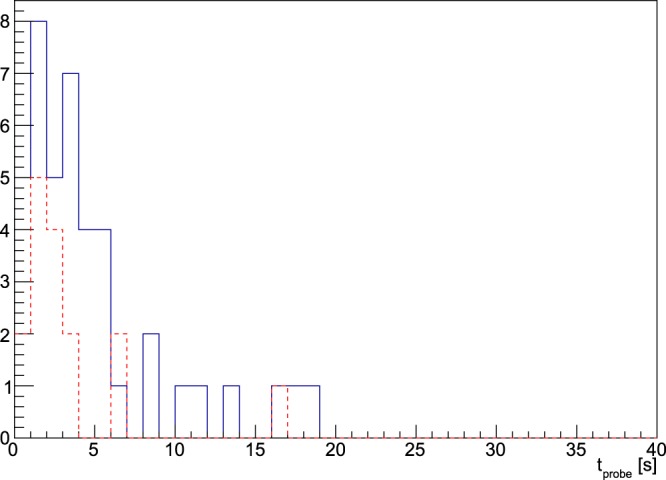
Distribution of ${t}_{probe}$ needed to be able to discriminate with sufficient accuracy the tumor. The statistical criterion used and the real case scenario considered are detailed in the text. The blue (continue) line represents lymph nodes, while the red (dotted) line represents local lesions.

**Figure 6:**
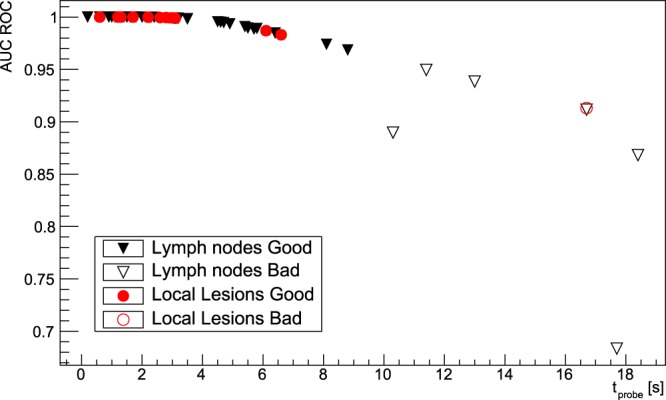
Area Under Curv of ROCs as a function of the minimum ${t}_{probe}$ calculated for each of the 58 lesions. In addition to the usual division among local lesions and lymph nodes, points are also classified (“Good” or “Bad”) according to the ROC sensitivity test described in the text, having fixed a probing time of 3 s and requiring $AUC > 0.95$.

In Fig. 5 the detecting times needed for the probe to discriminate the tumor from the surrounding healthy tissue, as described earlier in the text, are shown in an histogram, while in Fig. 6 the correlation between the ROC-AUC and the minimum time is reported.

## Discussion

SUVs are found as foreseen to differ significantly between the lesions, ranging from less than 2 in the lowest cases up to about 20. No particular difference appears to be present if the lesion is local or a lymph node, since the two populations seem to show analogous mean uptake.

A similar behaviour is exhibited by TNRs, that range from a minimum value of about 1.5 to a maximum value of above 60, with only a slight, non relevant, preference of lymph-nodes for higher values.

The vast majority of cases seem to show good TNR, of the same order of magnitude of those found for DOTATOC in case of meningioma, glioma^5^ and Neuro Endocrine Tumors (NET)^6^. However, while in those cases the efficacy of the technique is boosted by the pure ${\beta }^{-}$ emission of ^90^Y-DOTATOC, with substantially no *γ* contamination, in case of ^68^Ga-PSMA the presence of the abundant photon flux due to positron annihilation leads to a remarkable worsening in the quality of the ratio between counting rate on the tumor and on healthy tissue. It is to be noted that ^90^Y-PSMA would be the optimal tracer, but its use in clinics is still not established^18^.

Figure 5 suggests that a probing time ${t}_{probe}\sim 5$ is enough for the great majority of lesions to be identified accurately. These times, despite being considerably longer than those in case of pure ${\beta }^{-}$-RGS^6^, seem however to be compatible with an application scenario, especially considering that there is a “reaction time” of the surgeon that is estimated to be of the order of few seconds, independently from the probe signal.

The ROC sensitivity test described above suggests that the technique has very good discriminating power for the vast majority of cases, since from Fig. 3 (bottom) it stems that almost all the considered lesions have an AUC of more than $\mathrm{80 \% }$ with a reasonable ${t}_{probe}=3\,{\rm{s}}$. Setting a conservative cut at $AUC\ge 95 \%$ identifies “badly discriminated” cases, which represent lesions characterised by very low TNR, that are those in which the applicability of this technique is expected in fact to be more difficult and therefore cluster close to the bisector in Fig. 4. These cases correspond to values of ${t}_{probe}$ exceeding severals tens of seconds (see Fig. 5), for which this technique to be applicable would require a greater activity to be administered to the patient, with all the associated consequences in terms of radio-protection issues, both for the patient and for the medical personnel.

However it is crucial to highlight that the application protocol of *β*-RGS foresees a preliminary PET image with ^68^Ga-PSMA precisely to demonstrate that the lesion of interest shows a TNR good enough to make the patient a possible candidate for such a technique.

As far as radio protection issues are concerned, same considerations standing for proposed ${\beta }^{+}$-RGS techniques^19^ hold also in this case. Moreover, it has to be stressed that ^68^Ga has an mean lifetime that is approximately half of the one of ^18^F. This means that the use of the former would imply a much reduced radiation load both to the patient and to the medical staff.

It is finally to be noted that the current estimate is made with a detector optimised for ^90^Y and that improvements can be foreseen by either increasing the electron efficiency or decreasing the sensitivity to photons, for instance with the use a solide state *β* detector.

## Conclusions

The goal of this study was to evaluate the possibility to use the *β* probe we have developed for *β*-RGS with a high energy *β*^+^ emitting radio pharmaceutical, exploiting the high sensitivity of the detector, in order to extend the application cases of such a technique.

To this aim, we evaluated the uptake of ^68^Ga-PSMA in prostatic cancer recurrences in a retrospective study on PET images of 45 patients at *Policlinico Sant*’ *Orsola Malpighi* in Bologna.

Despite the expected variability among patients, both in terms of SUV (median 4.1, IQR 3.0–6.1) and TNR (median 9.4, IQR 5.2–14.6), the majority of cases have been found to be compatible with the application of the proposed technique in a reasonable scenario of injected activity and probing time. There are however some cases in which the technique to be effective would require either a grater amount of administered activity or a longer probing time.

The main result of this study is that most cases of prostatic cancers showing uptake for ^68^Ga-PSMA are good candidates for *β*-RGS. The preliminary PET image with ^68^Ga-PSMA, foreseen in the application protocol, would however allow to know in advance if the considered patient is a good candidate or not, also allowing to tailor the amount of activity to inject to the particular uptake.

To this aim, *ex*-*vivo* tests on excised tumor specimens are needed and foreseen for the next future, in order to assess the precise criteria to discriminate patients eligible for this RGS technique.

On the other hand, studies are being carried out to investigate possible novel detecting techniques to be used in the probe, for example focusing on solid state sensors (CMOS). This development of the instrument could allow to lower significantly the energy threshold for detecting electrons, thus improving the efficacy in an application case like the one discussed in this study.

## References

[1] Tsuchimochi M, Hayamaand K (2013). Intraoperative gamma cameras for radioguided surgery: Technical characteristics, performance parameters, and clinical application. Phys. Med..

[2] Schneebaum Schlomo, Even-Sapir Einat, Cohen Meir, Shacham-Lehrman Hedva, Gat Andrea, Brazovsky Eli, Livshitz Gennady, Stadler Jona, Skornick Yehuda (1999). Clinical applications of gamma-detection probes – radioguided surgery. European Journal of Nuclear Medicine.

[3] Solfaroli Camillocci, E. *et al*. A novel radioguided surgery technique exploiting *β*^−^ decays. *Sci Rep*. **4** (2014). 10.1038/srep04401 PMC3960579 24646766

[4] Russomando A (2016). An intraoperative beta- detecting probe for radio-guided surgery in tumour resection. IEEE-TNS.

[5] Collamati F (2015). Toward radioguided surgery with *β*- decays: uptake of a somatostatin analogue, dotatoc, in meningioma and high-grade glioma. Journal of Nuclear Medicine.

[6] Collamati F (2015). Time evolution of dotatoc uptake in neuroendocrine tumors in view of a possible application of radioguided surgery with *β*- decay. Journal of Nuclear Medicine.

[7] Camillocci ES (2016). First *ex vivo* validation of a radioguided surgery technique with *β*-radiation. Physica Medica: European Journal of Medical Physics.

[8] Mancini-Terracciano C (2017). Feasibility of beta-particle radioguided surgery for a variety of “nuclear medicine” radionuclides. Physica Medica: European Journal of Medical Physics.

[9] Graham, M. M., Gu, X., Ginader, T., Breheny, P. & Sunderland, J. 68ga-dotatoc imaging of neuroendocrine tumors: A systematic review and meta-analysis. *Journal of Nuclear Medicine* jnumed–117 (2017). 10.2967/jnumed.117.191197 PMC6944175 28280220

[10] Eiber M (2017). Prostate-specific membrane antigen ligands for imaging and therapy. Journal of Nuclear Medicine.

[11] Maurer T (2015). Prostate-specific membrane antigen–radioguided surgery for metastatic lymph nodes in prostate cancer. European urology.

[12] Maurer T (2016). Psma theranostics using pet and subsequent radioguided surgery in recurrent prostate cancer. Clinical genitourinary cancer.

[13] Camillocci ES (2017). Intraoperative probe detecting β- decays in brain tumour radio-guided surgery. Nuclear Instruments and Methods in Physics Research Section A: Accelerators, Spectrometers, Detectors and Associated Equipment.

[14] Fendler WP (2017). 68 ga-psma pet/ct: Joint eanm and snmmi procedure guideline for prostate cancer imaging: version 1.0. European journal of nuclear medicine and molecular imaging.

[15] Angelone M (2014). Properties of para-terphenyl as detector for alpha, beta and gamma radiation. IEEE Transactions on Nuclear Science.

[16] Bocci, V. *et al*. The ardusipm a compact trasportable software/hardware data acquisition system for sipm detector. *IEEE Nuclear Science Symposium and Medical Imaging Conference* (*NSS*/*MIC*) 1–5, 10.1109/NSSMIC.2014.7431252 (2014).

[17] Ferrari, A. *et al*. Fluka: a multi particle transport code. Tech. Rep. CERN-2005-10, INFN/TC05/11, SLAC-R-773 (2009).

[18] Bander NH (2005). Phase i trial of 177lutetium-labeled j591, a monoclonal antibody to prostate-specific membrane antigen, in patients with androgen-independent prostate cancer. Journal of Clinical Oncology.

[19] Bonzom S (2007). An intraoperative beta probe dedicated to glioma surgery: Design and feasibility study. IEEE Transactions on Nuclear Science.

